# Supplementary material for: Causal interplay between lactose intolerance and gut microbiota: a combined bidirectional Mendelian randomization and in vivo validation study
Source: Front Nutr. 2026 Jun 1;13:1803337. doi: 10.3389/fnut.2026.1803337 (PMC13265576; doi:10.3389/fnut.2026.1803337)
Supplement: Supplementary file 1 [file Data_Sheet_1.zip › supplementary materials/Forward/leave one out cross validation/ebi-a-GCST90027476.finngen_R12_E4_LACTONAS.pdf]

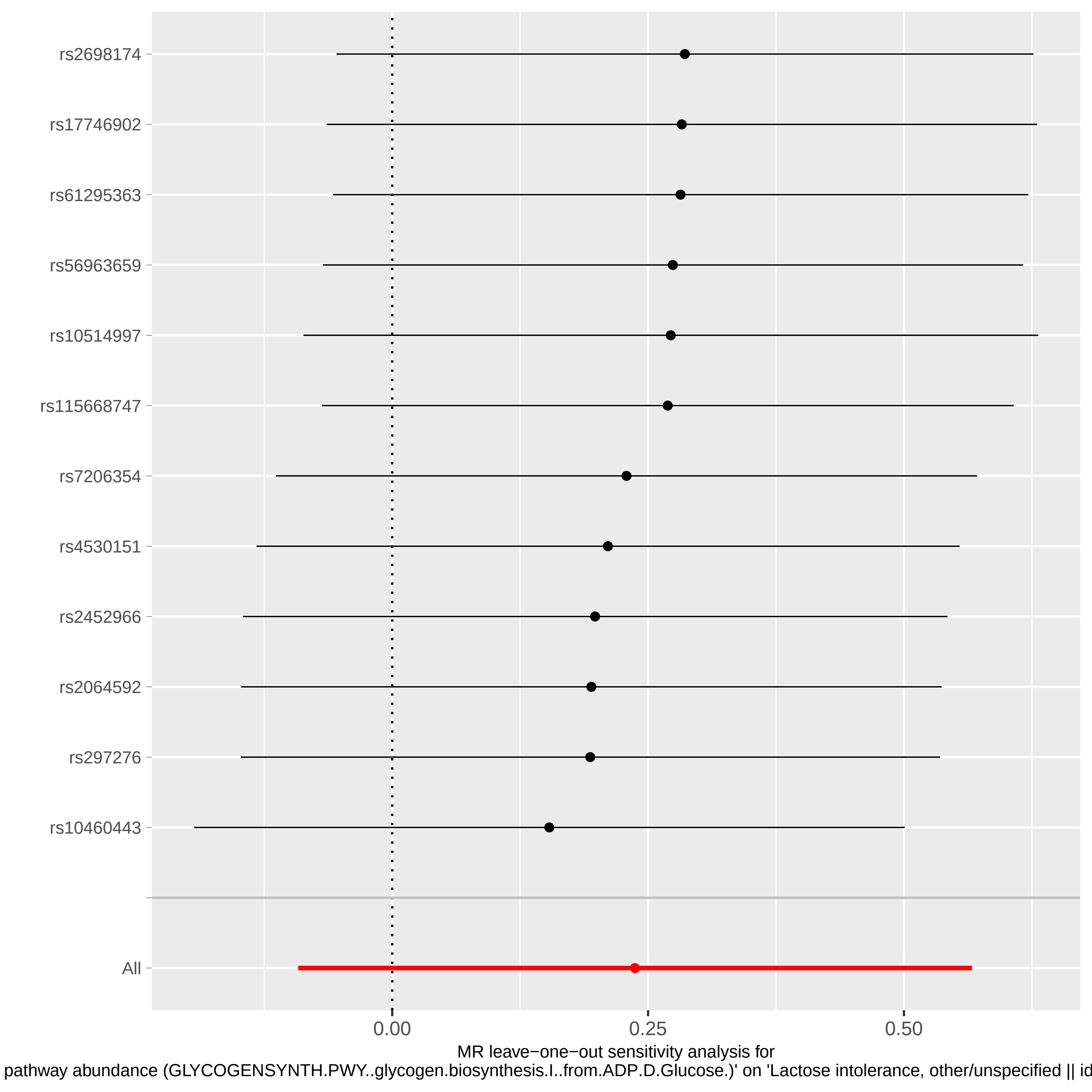

rs2698174
rs17746902
rs61295363
rs56963659
rs10514997
rs115668747
rs7206354
rs4530151
rs2452966
rs2064592
rs297276
rs10460443
All
0.00
0.25
0.50
MR leave−one−out sensitivity analysis for
pathway abundance (GLYCOGENSYNTH.PWY..glycogen.biosynthesis.I..from.ADP.D.Glucose.)' on 'Lactose intolerance, other/unspecified || id
